# The impact of gender bias in cardiothoracic surgery in Europe: a European Society of Thoracic Surgeons and European Association for Cardio-Thoracic Surgery survey

**DOI:** 10.1093/ejcts/ezac034

**Published:** 2022-01-29

**Authors:** Cecilia Pompili, Isabelle Opitz, Leah Backhus, Gunda Leschber, Giulia Veronesi, Olivia Lauk, Nuria Novoa, Niccolo’ Daddi, Indu Deglurkar, Julie Cleuziou, Anna Lena Emrich, Francesca D’Auria, Jolanda Kluin

**Affiliations:** 1 Section of Patient Centred Outcomes Research (PCOR), University of Leeds, Leeds, UK; 2 Leeds Teaching Hospital NHS Trust, Leeds, UK; 3 University Hospital Zurich, Zurich, Switzerland; 4 Stanford University Hospital, Palo Alto, CA, USA; 5 Berlin, Germany; 6 Ospedale San Raffaele, Milan, Italy; 7 University Hospital of Salamanca, Salamanca, Spain; 8 IRCCS Azienda Ospedaliera Universitaria di Bologna, Bologna, Italy; 9 University Hospital of Wales, Cardiff, UK; 10 German Heart Center Munich, Munich, Germany; 11 Medical University of South Carolina, Charleston, SC, USA; 12 University Medical Center Mainz, Mainz, Germany; 13 San Giovanni di Dio Ruggi d’Aragona University Hospital of Salerno—Schola Medica Salernitana, Salerno, Italy; 14 Academic Medical Center, Amsterdam, Netherlands

**Keywords:** Cardiothoracic surgery, Thoracic surgery, Gender bias, Female leadership, Mentorship, Professional life

## Abstract

**OBJECTIVES:**

The European Society of Thoracic Surgeons and the European Association for Cardio-Thoracic Surgery designed a questionnaire to assess the impact of gender bias on a cardiothoracic surgery career.

**METHODS:**

A 46-item survey investigating gender bias was designed using online survey software from December 2020 to January 2021. All European Society of Thoracic Surgeons and European Association for Cardio-Thoracic Surgery members and non-members included in the mailing lists were invited to complete an electronic survey. Descriptive statistics and a comparison between gender groups were performed.

**RESULTS:**

Our overall response rate was 11.5% (1118/9764), of which 36.14% were women and 63.69% were men. Women were more likely to be younger than men (*P* < 0.0001). A total of 66% of the women reported having no children compared to only 19% of the men (*P* < 0.0001). Only 6% of women vs 22% of men were professors. More women (72%) also reported never having been a formal mentor themselves compared to men (38%, *P* < 0.0001). A total of 35% of female respondents considered leaving surgery because of episodes of discrimination compared to 13% of men; 67% of women said that they experienced being unfairly treated due to gender discrimination. Of the male surgeons, 31% reported that they were very satisfied with their career compared to only 17% of women (*P* < 0.0001).

**CONCLUSIONS:**

Women in cardiothoracic surgery reported significantly high rates of experiences with bias that may prevent qualified women from advancing to positions of leadership. Efforts to mitigate bias and support the professional development of women are at the centre of newly formed European committees.

**Figure:**
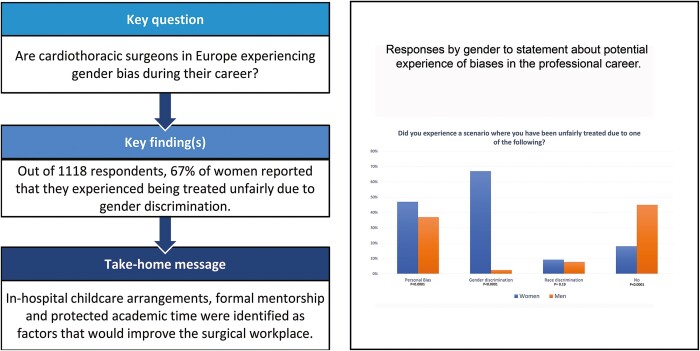

## INTRODUCTION

Despite the increasing proportion of women applying to European medical schools, relatively few women are in leadership positions, and several recent publications have highlighted many factors that could contribute to gender inequality in cardiothoracic surgery [1, 2]. However, there are some data to support a change in this landscape. As demonstrated in the USA, targeted efforts to support women surgeons have been linked to success, as evidenced by the improved retention and the reduced attrition along the training pipeline in cardiothoracic surgery [3]. The national and international societies have recently shown increased interest in addressing the gender disparity at higher institutional levels [4, 5]. In Europe, the process may be more complicated, considering the differences in cultures, healthcare systems and training programmes, but nonetheless it is an effort worth making [6].

The European Society of Thoracic Surgeons (ESTS) and the European Association for Cardio-Thoracic Surgery (EACTS) designed a questionnaire to assess surgeon demographics and the impact of gender bias on a career in cardiothoracic surgery.

The findings represent a snapshot of the members of the 2 societies. The results of this survey will help in identifying possible initiatives to support the next generations in pursuing a career in cardiothoracic surgery.

## MATERIALS AND METHODS

### Ethics statement

All ESTS (*N*: 1422) and EACTS (*N*: 8339) members and non-members included in the mailing lists of the societies that gave their consent to be contacted received an email inviting them to complete an electronic survey. The survey was open from 4 December 2020 through 17 January 2021. Responses were anonymous and were collected through a link to a commercially available platform (www.surveymonkey.com). This study was approved by both the ESTS and the EACTS councils. Two reminders were sent during this period via email prior to study closure.

### Survey design

A 46-item survey investigating gender bias was designed using online survey software and distributed with an introductory letter explaining the purposes of the survey (Supplementary material).

There were no exclusion criteria. A social media campaign was also implemented to disseminate the survey (Twitter and LinkedIn) and improve the response rate. All responses were voluntary and anonymous.

The questionnaire was designed by a team of 14 cardiothoracic surgeons belonging to both societies (80% women and 20% men) and subsequently submitted for revisions and approvals to the ESTS (11/14 men) and EACTS (12/13 men) Board of Directors. The developer team had experience in questionnaire design methodology, and the number and length of the questions was based on agreement of the members in the developer team.

The questionnaire was designed to elicit objective data regarding the respondents’ demographics, training and professional information; personal and/or family status (carer’s responsibilities, children); parental leave availability; career choice decisions; and perception of the specialty regarding gender bias, access to leadership positions and other discriminatory factors that may have affected the respondents’ careers.

Participants were asked on a 5-point Likert-like scale to agree or disagree with various statements regarding the role of gender in various well-known difficult scenarios or perceived situations. Statements describing the influence of potential barriers to a surgical career for women were also rated on a Likert-like scale.

### Statistical analyses

Normal distribution of numeric variables was assessed by the Shapiro–Wilk test. Numeric variables with normal distribution were compared using the unpaired *t*-test, whereas those without normal distribution were compared using the Mann–Whitney *U*-test. Categorical variables were compared using the χ^2^ test or the Fisher’s exact test (if the number of observations was <5).

Categorical data were expressed as counts and percentages. Statistical analyses were performed using Stata software (Stata Corp, College Station, TX, USA) with significance at an alpha level of 0.05.

## RESULTS

### Participant demographics

**Figure 1:**
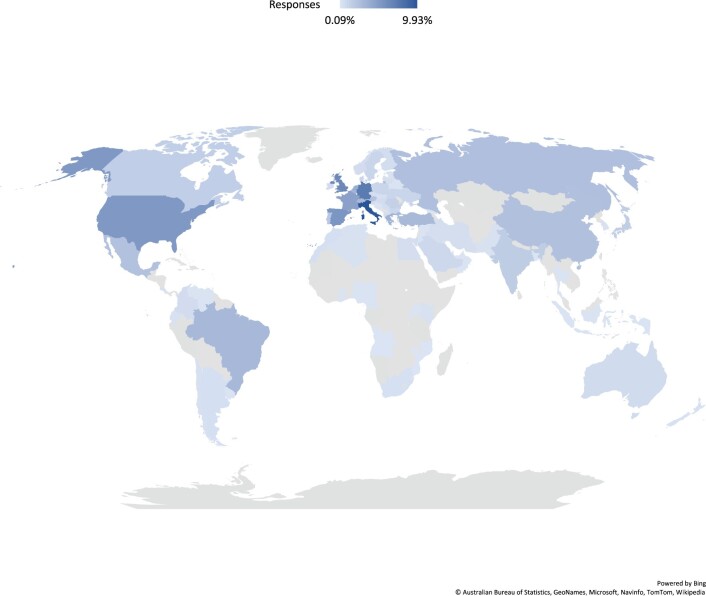
Geographical location of the respondents.

Our overall response rate was 11.5% (1118/9764). Of the 1118 total survey respondents, 36.14% were women and 63.69% were men (Table 1). Most of the respondents practiced cardiothoracic surgery in an academic hospital/university or government medical centre setting. Society membership among respondents was 60% declaring a primary EACTS membership compared to 35% declaring primary ESTS membership. Geographic location is shown in Fig. 1.

**Table 1:** Demographics of respondents

	Total		Female		Male		*P*-Value
	%	*N*	%	*N*	%	*N*	
Gender							
Female	36.14	404					
Male	63.69	712					
Other	0.18	2					
Professional membership							
ESTS	34.79	389					
EACTS	60.38	675					
STS	19.23	215					
AATS	8.59	96					
Other (please specify)	33.90	379					
Current practice setting							
Private—hospital employed	14.31	160					
Private—other	3.22	36					
Private—solo practice	2.15	24					
Academic clinical (primary)	42.40	474					
Academic research (primary)	3.40	38					
Government-run hospital	31.31	350					
Other (please specify)	3.22	36					
Years of training							
1	3.76	42					
2	7.42	83					
3	10.55	118					
4	8.32	93					
5	23.97	268					
>5	45.97	514					
Age							<0.0001
Under 30	6.44	72	11.63	47	3.51	25	
30–39	34.26	383	54.20	219	23.03	164	
40–49	23.35	261	20.54	83	24.85	177	
50–59	21.02	235	10.64	43	26.82	191	
60–69	11.36	127	2.90	12	16.15	115	
70+	3.58	40	0.00	0	5.61	40	
Number of years post-training							<0.0001
Currently in training	15.38	172	27.72	112	8.42	60	
0–5 years	22.00	246	34.15	138	15.16	108	
6–9 years	11.09	124	14.35	58	9.12	65	
10–19 years	21.20	237	14.85	60	24.85	177	
20–29 years	17.71	198	6.43	27	23.87	170	
≥30 years	12.61	141	2.22	9	18.53	132	
Current position							<0.0001
Trainee/fellow	23.52	263	40.59	164	13.30	95	
Consultant surgeon	48.03	537	36.63	148	40	285	
Assistant professor	8.86	99	8.41	34	8.70	62	
Associate professor	9.75	109	6.43	26	11.37	81	
Professor	15.56	174	6.18	25	21.91	156	
Retired/not currently in practice	1.16	13	0.24	1	1.12	8	
Other (please specify)	6.08	68	1.23	5	0.56	4	
Primary area of practice (>50%)							0.101
Congenital cardiothoracic surgery	8.50	95	8.90	36	8.14	58	
Adult cardiac surgery	48.84	546	45.79	185	50.70	361	
General thoracic surgery	39.27	439	40.59	164	38.48	274	
Other (please specify)	3.40	38	4.70	19	2.66	19	
Training outside your country							<0.0001
Yes	63.51	710	53.40	216	69.10	492	
No	36.49	408	46.53	188	30.89	220	
Working pattern							0.849
Full time	94	702	93	270	94	430	
Part time	6	47	7	19	6	28	
Marital status							<0.0001
Single (never married or never in a civil partnership)	16.64	176	36.87	139	5.44	37	
Married/in a civil partnership	70.89	750	47.21	178	83.90	570	
Separated	4.16	44	3.44	13	4.56	31	
Widowed	0.95	10	1.06	4	0.88	6	
Co-habitation/domestic partnership	6.33	67	9.54	36	4.56	31	
Prefer not to answer	1.04	11	1.85	7	0.58	4	
Number of children							<0.0001
0	35.54	376	65.78	248	18.85	128	
1	16.26	172	12.99	49	18.11	123	
2	28.07	297	13.52	51	36.08	245	
3	13.89	147	6.36	24	17.96	122	
More than 3	6.24	66	1.32	5	8.93	61	
Carer responsibility							<0.0001
Primary carer of a child still necessitating daily support	26.18	277	22	82	28	193	
Primary carer of a child 14–18 years old, mostly independent	7.28	77	3.44	13	9.42	64	
Primary carer or assistant for an older person or people (65 years and over)	5.01	53	7.16	27	3.82	26	
Primary carer of a child of >18 years	7.84	83	3.71	14	10	69	
None of the above	53.69	568	64	241	48	327	
Leadership position held							
Lead or head of department	31.38	332	11	40	43	291	<0.0001
President of a cardiothoracic society	7.84	83	3.1	12	10	71	<0.0001
Chair of an organization/body	14.37	152	7.7	29	18	123	<0.0001
Executive committee of a society/association	17.30	183	7.9	30	23	153	<0.0001
Board member in your organization	23.44	248	12	46	30	202	<0.0001
Research director	12.10	128	5	19	16	108	<0.0001
Trainee lead	26.84	284	19	71	31	213	<0.0001
None of the above	38.00	402	60	228	26	174	<0.0001
Other leadership roles (please specify)	7.09	75	6.6	25	7.3	50	0.59
Have you been a formal mentor?							<0.0001
Yes	49.62	525	28	105	62	417	
No	49.91	528	72	266	38	259	
Have you had a formal mentor?							<0.0001
Yes	50.85	538	40	123	56	381	
No	49.43	523	60	224	44	296	
How many peer-reviewed first or last/senior author publications do you have to your credit?							<0.0001
0–5	44.57	369	62	201	33	168	
6–10	17.75	147	19	60	17	86	
11–20	11.59	96	9	29	13	67	
21–50	11.11	92	4	13	16	78	
>50	14.98	124	6	21	21	103	
Have you applied for external grant funding for research as a Principal Investigator? If so, how many times							<0.0001
3 or less	29.71	246	27.50	89	31	156	
Over 3	16.06	133	9.50	31	20	102	
Never	54.23	449	63	204	49	244	

AATS: American Association for Thoracic Surgery; EACTS: European Association for Cardio-Thoracic Surgery; ESTS: European Society of Thoracic Surgeons; STS: Society of Thoracic Surgeons

Women in cardiothoracic surgery were more likely to be younger than men (*P* < 0.0001), most of them reporting to be 30–39 years of age; the age distribution among men was spread equally from 30 to 69 years. A similar trend was also observed in terms of the number of years post-training: Most female (34%) respondents were in their first 5 years after completion of training compared to 24% of male respondents being 20–29 years out from training (*P* < 0.0001). Ninety-three percentage of women reported working full time.

Accordingly, most women were currently in training positions or fellowships compared to men being in their consultant/staff roles. Somewhat over 8% of female and male respondents were assistant professors, whereas only 6% of female vs 22% of males were full professors. More male participants (43%) reported to be head of the department compared to women. More male participants had experiences of working outside their countries compared to women (69% vs 53%; *P* < 0.0001).

When asked about their personal life, most of the women reported being married or in a civil partnership (47%) whereas 37% reported being single compared to 84% of men who reported being married. Interestingly, 66% of women reported having no children compared to only 19% of men (*P* < 0.0001). This result affects the answers to the questions regarding carer responsibilities: 37% of male and 25% of female respondents reported to be the primary carer of a child under 18 years of age.

### Mentorship and professional experience

More female surgeons reported not having had a formal mentor (60%) compared to male respondents (44%, *P* < 0.0001). More women (72%) also reported never having been a formal mentor themselves compared to men (38%, *P* < 0.0001). There was also a significant difference in the proportion of respondents who reported having participated in formal leadership or mentorship programmes (women: 37% vs men: 56%; *P* < 0.0001). In terms of academic output and grant funding submissions, female respondents reported lower rates compared to their male counterparts.

### Gender bias in professional life

Thirty-five percentage of female respondents indicated that they considered leaving surgery because of episodes of discrimination, compared to only 13% of men (Table 2). Sixty-seven percentage of women indicated that they experienced being unfairly treated due to gender discrimination compared to only 2.5% of male respondents. Few men reported postponing having children (14%) compared to nearly half of women despite their younger reported ages (44%). Respondents were asked to rate their level of satisfaction in their professional careers. More than one-third of male surgeons (31%) reported that they were very satisfied, whereas women reported much less satisfaction (17%, *P* < 0.0001). The question ‘How often do you feel your gender has influenced your interactions negatively with others in your professional environment?’ was answered with ‘very much’ by 24% and with ‘somehow’ by 44% of women whereas the corresponding percentages in answers by male surgeons were 1% and 13%, respectively. Fifty-two percentage of males answered the aforementioned question with ‘not at all’ compared to only 7% of female surgeons. Interestingly, women declared they felt less valued in their current work environment compared to the men (*P* < 0.0001).

**Table 2:** Discrimination experiences

	Total		Female		Male		*P*-value
	%	*N*	%	*N*	%	*N*	
Have you ever considered leaving surgery because of discrimination?							<0.0001
Yes often	6.64	55	13	41	3	14	
Yes sometimes	21.62	179	35	114	13	65	
No	51.57	427	41	133	58	293	
Not relevant—I have not experienced any discrimination	18.84	156	9	30	25	125	
I do not know	1.33	11	2	6	1	5	
Did you experience a scenario where you have been unfairly treated due to one of the following							
Personal bias	40.94	339	47	151	37	188	<0.0001
Gender discrimination	27.66	229	67	216	2.5	13	<0.0001
Race discrimination	8.33	69	9.25	30	7.76	39	0.19
No	38.16	316	18	59	45	255	<0.0001
Prefer not to answer	4.47	37	3.39	11	5.17	26	
How do you think/experienced childbearing will/did affect your professional life?							<0.0001
Training will take/took longer due to pregnancy/parental leave	42.15	349	30	97	50	251	
Had to stop working/training due to pregnancy	15.58	129	16	53	15	76	
Will postpone/postponed pregnancy to a later time	25.97	215	44	143	14	71	
Will have/had the opportunity to use the time for academic work	16.30	135	10	31	21	104	
Please indicate level of satisfaction in your professional career							<0.0001
Very satisfied	25.97	215	17	56	31	158	
Satisfied	47.95	397	47	153	48	243	
Neither satisfied nor dissatisfied	15.94	132	19	60	14	72	
Dissatisfied	8.45	70	14	45	5	25	
Very dissatisfied	1.69	14	3	10	0.80	4	
How often do you feel your gender has influenced your interactions negatively with others in your professional environment?							<0.0001
Very much	10.39	86	24	79	1.39	7	
Somehow	25.12	208	44	142	13	66	
Not much	26.09	216	23	74	28	142	
Not at all	34.78	288	7.09	23	52	263	
Undecided	3.62	30	1.80	6	4.78	24	
How valued do you feel in your current work environment?							<0.0001
Extremely valuable	16.06	133	10	33	20	100	
Very valuable	39.13	324	31	99	45	224	
Somewhat valuable	33.45	277	41	133	28	143	
Not so valuable	8.09	67	13	43	4.78	24	
Not at all valuable	3.26	27	4.93	16	2.19	11	

### Gender bias

Participants in the survey were asked to reflect on the extent of gender bias within our discipline (Table 3). The responses from women in cardiothoracic surgery indicated that taking time off for parental leaves for women is still considered an issue in cardiothoracic surgery. Although the responses of male and female cardiothoracic surgeons were more concordant concerning some of the more generic challenges related to female work–life integration (i.e. both agreed with ‘Female surgeons incur more disadvantages by having a family than male surgeons’ and ‘Some surgeons do not understand the difficulty of female surgeons have balancing work and family/personal life’), they responded markedly differently to most of the other more specific aspects (i.e. ‘A female surgeon can expect resentment if she takes maternity leave’ or ‘Most surgeons in leadership are supportive of female surgeons who want to balance their family and career lives’).

**Table 3:** Responses by women and men in cardiothoracic surgery to gender bias statements

	Male %	Female %
A female surgeon can expect resentment if she takes parental leave		
Agree/strongly agree	28	63
Disagree/strongly disagree	41	16
Neutral	31	21
Most surgeons in leadership are supportive of female surgeons who want to balance their family and career lives		
Agree/strongly agree	47	14
Disagree/strongly disagree	26	68
Neutral	28	18
Surgeons who bring up issues about balancing family and career usually would be supported		
Agree/strongly agree	48	55
Disagree/strongly disagree	25	22
Neutral	27	23
Most surgeons would feel comfortable and supportive of a female chairperson		
Agree/strongly agree	51	22
Disagree/strongly disagree	17	56
Neutral	32	22
Informal conversations following a meeting often exclude female colleagues		
Agree/strongly agree	8	37
Disagree/strongly disagree	77	40
Neutral	16	23
Male and female surgeons have equal income		
Agree/strongly agree	64	30
Disagree/strongly disagree	18	55
Neutral	18	15
Male surgeons are as likely to discuss academic issues with a female colleague		
Agree/strongly agree	70	36
Disagree/strongly Disagree	10	30
Neutral	20	34
Some surgeons do not understand the difficulty female surgeons have balancing work and family/personal life		
Agree/strongly agree	70	84
Disagree/strongly disagree	14	6
Neutral	16	10
A male surgeon can expect resentment if he takes parental leave		
Agree/strongly agree	43	36
Disagree/strongly disagree	30	39
Neutral	27	25
Female surgeons incur more disadvantages by having a family than male surgeons		
Agree/strongly agree	67	88
Disagree/strongly disagree	17	6
Neutral	16	5
Female surgeons who have taken time off to have children are considered just as committed as those who have not taken time off		
Agree/strongly agree	44	21
Disagree/strongly disagree	29	59
Neutral	27	20

Women more strongly disagree that most of the surgeons would consider a female chairperson a supportive and comfortable figure, compared to the men.

Furthermore, responses from men and women diverged significantly with respect to workplace treatment (e.g. ‘Informal conversations following a meeting often exclude female colleagues’ or ‘Male and female surgeons have equal income’).

**Figure 2:**
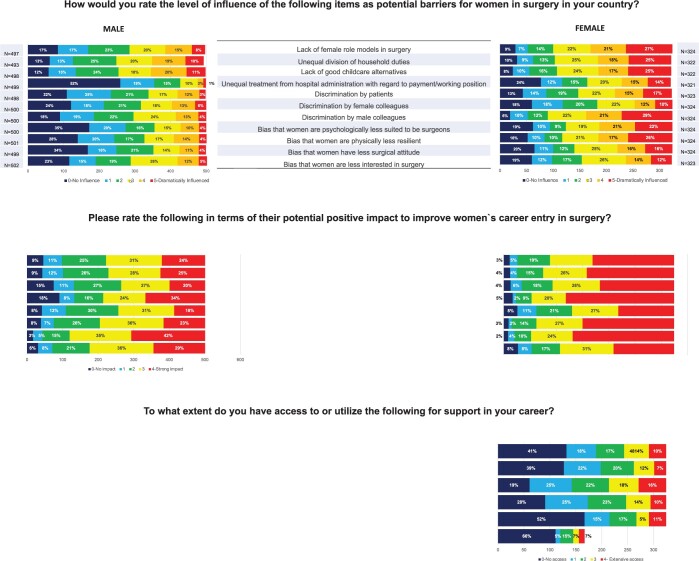
Stacked plots of responses by women and men in cardiothoracic surgery to statements about potential barriers to career progression.

In addition, substantial differences in answers from women and men are seen when they are asked to identify the potential barriers for women in surgery (Fig. 2). More than half of the women indicated that every item except discrimination by female colleagues could be a potential barrier for women in surgery, whereas men rated all items to be less important barriers. Women identified not only discrimination within the surgical field or institutions as potential barriers but also discrimination by patients.

**Figure 3:**
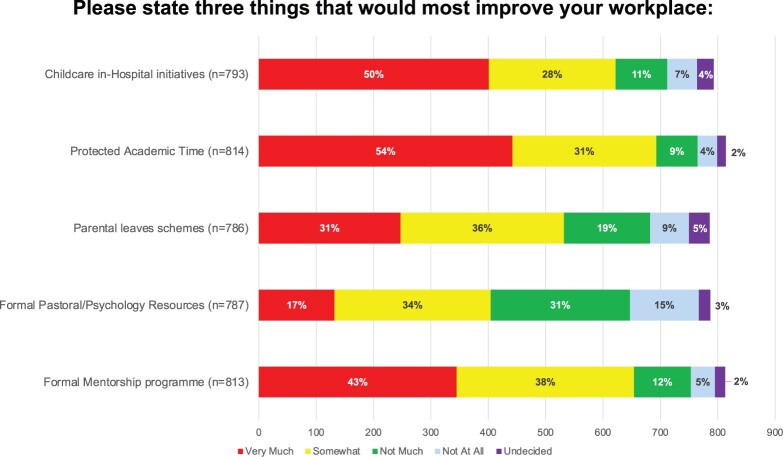
Stacked plots of responses by both women and men in cardiothoracic surgery to factors that will mostly improve their workplace.

When participants were asked to identify 3 main factors that would improve their workplace, in-hospital childcare arrangements, formal mentorship and protected academic time were found to play a significant role (Fig. 3).

## DISCUSSION

Women in cardiothoracic surgery report significantly high rates of experiences with bias that may prevent qualified women from advancing in positions of leadership. Poor gender diversity at the leadership level also has negative consequences for the quality of work being done by national and international organizations. Our survey clearly demonstrated that across the 2 main European cardiothoracic associations, there is still a lower percentage of women in leadership roles or positions of influence. Although some of this difference may be influenced by sampling bias, with the majority of female respondents being at a more junior level compared to the male respondents, it is also indicative of the fact that women remain clustered at the junior ranks of most of the institutions to which they belong. The results confirm the hypothesis that women and men have significant differences with regard to their lived experiences and that some of these differences translate into higher attrition for women. Most of the women surveyed experienced gender discrimination and reported to have thought about leaving our speciality because of this discrimination.

The Society of Thoracic Surgeons and Women in Thoracic Surgery (WTS) recently surveyed and obtained responses from 633 members to investigate the extent of gender bias within our discipline [1]. Similar to our results, they found that the perception of gender bias varied greatly between male and female respondents. The role of societies like the WTS is recognized as groundbreaking, and these societies have already demonstrated their potential in providing mentorship, training and networking opportunities for female surgeons. The recently formed ESTS Women in General Thoracic Surgery Committee and the EACTS Women in Cardiothoracic Surgery Committee will lead the campaign to inspire more women to fulfil their surgical ambitions.

Furthermore, from the USA, we have seen promising examples of positive changes. These changes have led to an increased representation among the Southern Thoracic Surgical Association [7] and also to higher success rates in the career progression of the WTS scholarship awardees [3], confirming the key role of these initiatives to enhance and support the progression of women’s careers.

The role of mentorship and sponsorship of women by women is critically important in achieving maximum career potential, as demonstrated in recent papers in various surgical specialities [8]. This approach plays a crucial role in countries where there is a lack of exposure of women from the earliest stages of their careers to female role models or mentors [9]. European societies can play an important role in providing support for women who commence a career but who might subsequently become discouraged and abandon it, unless they receive this positive support. A recent review of mentoring programmes and scholarships sponsored by the WTS demonstrated that participation in these structured programmes was associated with successful pursuit of career milestones at significantly higher rates, likely due to fostering of a supportive community for women trainees [3].

The percentage of full professors in surgery who are women is increasing at a rate disproportionately slower than the increases in female medical students and surgical residents [10]. In Europe, this situation may be very heterogeneous: not only does each country have a specific healthcare system but culturally the barriers to female progression can be enhanced and strengthened by stereotypes and lack of role models even outside medicine [6, 11–13].

Gender-based disparities in academic surgery have become the focus of recent surveys and articles; however, our overall knowledge of the situation of women in surgery actively involved in the 2 main European cardiothoracic societies is still limited. Our results showed that the gender disparity is more pronounced in academic medicine, especially when specific output results or grant applications are involved. The early exposure to research in medical school and residency has again a strong potential: A larger US national cohort study showed that the relation between participation in research during residency and future faculty appointment was stronger among women than among men [14]. Social media have an increasingly defined role in facilitating professional networking and academic dissemination, especially for women in thoracic surgery who lack direct same-sex mentorship exposure [15]. We may not rule out that this situation has played a role in the favourable improvement in female representation of authorship in our field [16].

Our study findings confirmed how complex the position of women in cardiothoracic surgery in Europe remains, demonstrating the need for education and change. First, most sources of perceived discrimination were gender specific, which seem to be reflected in the lower level of satisfaction in the professional career for women and a clear tendency of female respondents to consider postponing childbearing to a later time. The changing policies in many countries towards men’s involvement in early child-rearing have received little attention but should be emphasized. New family-friendly policies that encourage men as well as women to take career breaks, or work part time to share childcare, could have the indirect effect of encouraging their female partners to enter, and remain in, an academic career.

Furthermore, it is also clear that men’s consideration of the potential impact of gender bias differs consistently from what women reported. Discrimination from patients is mostly reported by female respondents: As reported across specialities, women physicians are often addressed as ‘nurse’ instead of ‘doctor’ or are introduced by their first name rather than by their title [17]. Societal awareness may play an important role in reducing gender bias inside hospitals. Empowering women from the beginning of their medical school through mentorship programmes or fellowships may help reduce the misconception reported in our findings that women are less interested, have less surgical attitude or are physically less resilient than men.

Our findings indicate that salary equity for female surgeons, access to onsite childcare support and more role models’ examples should have a positive impact on improving women’s career entry in surgery.

This work will expand the evidence behind the recent implementation of diversity and inclusion initiatives and educational activities in Europe. Research programmes and grant institutions equally increased the awareness by forming gender bias committees designed specifically to deal with this topic. Surgical societies like ESTS and EACTS have formed Women in Thoracic and Cardiothoracic Surgery committees to ensure and support a climate of respect and inclusion among members.

### Limitations

This study has some potential limitations that need to be considered when interpreting the results. Some survey respondents were from several countries beyond the European borders, so we may not rule out the fact that different socioeconomic situations and healthcare systems have had an impact on the participants’ responses. Our results may have been affected by the differences of the 2 groups (male and female) in terms of age: however, these differences reflect the glass ceiling effect that is still evident in our speciality, confirmed by the differences in representation of women among the ESTS trainee members compared to the active members (44% vs 19%).

Although our response rate is consistent with those of other electronic healthcare professionals surveys, it is low. Both the length of the questionnaire and the fact that it was sent electronically likely contributed to our low response rate. We did not have the data about the delivery status of these 2 major mailing lists or the actual membership status of the recipients, which limited our estimate of the sample size and response rate.

Furthermore, to our knowledge, there are no validated surveys for investigating specific gender bias in surgery. Recall bias is also a relevant limitation, particularly considering the percentage of senior participants in our survey.

Lastly, we left the definition of ‘formal mentor’, ‘formal leadership and mentorship programme’ and ‘unfairly treated’ to the members themselves.

## CONCLUSIONS

Our findings depicted a sobering situation in terms of the representation of women in cardiothoracic surgery as well as the issue of gender bias. Efforts to mitigate bias and support the professional development of women are at the centre of the newly formed European committees.

## SUPPLEMENTARY MATERIAL

Supplementary material is available at *EJCTS* online.

## ACKNOWLEDGMENT

The authors would like to thank all the colleagues who participated in the survey.

**Conflict of interest:** Cecilia Pompili reports consultancy for Medela, AstraZeneca (AZ) and Becton Dickinson outside the submitted work. Giulia Veronesi reports honoraria from Ab Medica, Intuitive Surgical for consultation and proctoring, outside the submitted work. Isabelle Opitz reports Roche and AZ: Speakers Bureau, Advisory Board for AZ and Merck and an institutional grant from Roche and Medtronic outside the submitted work. Gunda Leschber reports AZ: Speakers Bureau outside the submitted work.

## Data Availability Statement

All relevant data are within the manuscript and its supporting information files.

## Supplementary Material

ezac034_supplementary_data

## Notes

Presented at the 29th European Conference on General Thoracic Surgery, 20–22 June 2021.

Presented at the 35th EACTS Annual Meeting 13–16 October 2021 Barcelona, Spain.

## ABBREVIATIONS

EACTS: European Association for Cardio-Thoracic Surgery
ESTS: European Society of Thoracic Surgeons
WTS: Women in Thoracic Surgery
