# Supplementary material for: The impact of gender bias in cardiothoracic surgery in Europe: a European Society of Thoracic Surgeons and European Association for Cardio-Thoracic Surgery survey
Source: Eur J Cardiothorac Surg. 2022 Jan 29;61(6):1390–9. doi: 10.1093/ejcts/ezac034 (PMC9746891; doi:10.1093/ejcts/ezac034)
Supplement: ezac034_supplementary_data [file ezac034_supplementary_data.zip › FINAL survey version .pdf]

The ESTS/EACTS Gender Bias Workforce Survey

Please take a few moments to answer these important questions to help ESTS/EACTS understand the needs of Women in Cardiothoracic Surgery.

1. Please indicate your age group:

   a. <30
   b. 30-39
   c. 40-49
   d. 50-59
   e. 60-69
   f. ≥70

2. Gender

   a. Female
   b. Male
   c. Other

3. Number of years in practice post-training:

   a. Currently in training
   b. 0-5 years
   c. 6-9 years
   d. 10-19 years
   e. 20-29 years
   f. ≥30 years

4. Professional membership:

   a. ESTS
   b. EACTS
   c. STS
   d. AATS
   e. Other, please specify

5. Current Practice setting

   a. Private – Hospital employed
   b. Private – Other
   c. Private – Solo practice
   d. Academic Clinical (primary)
   e. Academic Research (primary)
   f. Government run Hospital
   g. Others:

6. What is your current position? Multiple choices allowed

    a. Trainee/Fellow
    b. Consultant Surgeon
    c. Assistant Professor
    d. Associate Professor
    e. Professor
    f. Emeritus
    g. Retired/not currently in practice
    h. Other: __________

7. Country of current practice

    Drop down list

8. What is your primary area of practice currently (>50%)?

    a. Congenital Cardiothoracic Surgery
    b. Adult Cardiac Surgery
    c. Adult Thoracic Surgery
    d. Other
       Please specify: Free text

9. How many years were you in Cardiothoracic training?

    Drop down list

10. Have you done any training outside of your home country?
    a. Yes
    b. No

11. If answered yes to 11, then which country?
    a. Dropdown (same as above)

12. Are you or have you been (select all that apply)

    a. Lead or Head of Department. Yes/ No
    b. President of a Cardiothoracic Society. Yes/No
    c. Chair of an Organization/body. Yes/No
    d. Executive Committee of a Society/ Association. Yes/No
    e. Board member in your Organization. Yes/No
    f. Research Director. Yes/No
    g. Trainee Lead
    h. Other leadership roles
       Please specify: Free text

13. Did you have a formal mentor (e.g assigned or elected with regular communication, advice, career planning) when you commenced independent practice?

i. Yes
j. No
If not, why not (free text)

14. Have you been a formal mentor to surgeons commencing independent practice?

k. Yes
l. No
If not, why not (free text)

15. Have you participated in any formal leadership training or mentoring programs either through your employer, institution or professional society?

m. Yes
If so, which (check all that apply):
1) Individual Hospital
2) Private
3) STS
4) AATS
5) ESTS
6) Other

a. No
If not, why not (free text)

16. Are there policies in your Institution for maternity leave?

a. Yes
b. No
c. I do not know

i. If yes, how long is maternity leave:

a. 4-12 weeks
b. 3-6 months
c. 6-12 months
d. 1 year

ii. If yes, what is the pay during leave?
a. Full salary for duration from employer
b. Partial salary for duration from employer
c. Full salary for duration from government
d. Partial salary for duration from government
e. Uncompensated

17. Are there policies in your Institution for paternity leave?
d. Yes
e. No
f. I do not know

ii. If yes, how long is maternity leave:

e. 4-12 weeks
f. 3-6 months
g. 6-12 months
h. 1 year

iii. If yes, what is the pay during leave?

a. Full salary for duration from employer
b. Partial salary for duration from employer
c. Full salary for duration from government
d. Partial salary for duration from government
e. Uncompensated

18. How many hours per week are you currently working (including research time)?

f. Full time, please specify average hours per week :
g. Part time, please specify average hours per week:

19. Does your Department offer part time or flexible work hours?

a. Yes
b. No
c. Only in exceptional cases
d. I do not know

20. Would you take up part time work or flexible work hours if offered?

a. Yes, if offered
b. Yes, if this would not have an effect on my current position
c. No, although it would not affect my current position
d. No, flexible working hours would be sufficient
e. never

21. Is research a condition of your job description?

a. Yes
b. No

i) If yes, do you have protected research time?

(1) Yes
(2) No

(i) If yes, how many hours/month are dedicated to research

22. Have you been interested in the academic pathway?

a. No
b. Yes
c. Yes, but no capacity due to family duties
d. Yes, but no support systems/mentor

23. How many peer-reviewed first or last/senior author publications do you have to your credit?
    a. 0-5
    b. 5-10
    c. 10-20
    d. 20-50
    e. >50

24. Have you applied for external grant funding for research as a Principle Investigator? If so, how many times:
    a. <3
    b. >3
    c. Never

25. Have you ever considered leaving surgery because of discrimination?
    a. Yes often
    b. Yes sometimes
    c. No
    d. Not relevant - I have not experienced any discrimination
    e. I don't know

26. Did you experience a scenario where you have been unfairly treated due to one of the following: (check all that apply)
    a. Personal Bias
    b. Gender discrimination
    c. Race discrimination
    d. Other, please specify
    e. No
    f. Prefer not to answer

27. Please indicate level of satisfaction in your professional career (Likert scale)

**Very much**	**Somewhat**	**Not much**	**Not at all**	**Undecided**

28. How valued do you feel in your current work environment? (Likert scale)

**Very much**	**Somewhat**	**Not much**	**Not at all**	**Undecided**

29. Please agree or disagree to the following statements:

A. Most surgeons would feel comfortable and supportive of a female chairperson.

Strongly Disagree	Disagree	Neutral	Agree	Strongly Agree

B. Female surgeons are less likely to have influence on departmental politics. In meetings people pay just as much attention when female surgeons speak as when male surgeons speak.

Strongly Disagree	Disagree	Neutral	Agree	Strongly Agree

C. Informal conversations following a meeting often exclude female colleagues.

Strongly Disagree	Disagree	Neutral	Agree	Strongly Agree

D. Male surgeons are as likely to discuss academic issues with a female colleague.

Strongly Disagree	Disagree	Neutral	Agree	Strongly Agree

E. Most surgeons in leadership are supportive of female surgeons who want to balance their family and career lives.

Strongly Disagree	Disagree	Neutral	Agree	Strongly Agree

F. Surgeons who bring up issues about balancing family and career usually would be supported.

Strongly Disagree	Disagree	Neutral	Agree	Strongly Agree

G. Some surgeons do not understand the difficulty female surgeons have balancing work and family/personal life.

Strongly Disagree	Disagree	Neutral	Agree	Strongly Agree

H. A female surgeon can expect resentment if she takes parental leave.

Strongly Disagree	Disagree	Neutral	Agree	Strongly Agree

I. A male surgeon can expect resentment if he takes parental leave.

Strongly Disagree	Disagree	Neutral	Agree	Strongly Agree

J. Female surgeons who have taken time off to have children are considered just as committed as those who have not taken time off.

Strongly Disagree	Disagree	Neutral	Agree	Strongly Agree

K. Female surgeons incur more disadvantages by having a family than male surgeons.

Strongly Disagree	Disagree	Neutral	Agree	Strongly Agree

L. Male and female surgeons have equal income.

Strongly Disagree	Disagree	Neutral	Agree	Strongly Agree

30. Are you:

a. Single (never married or never in a civil partnership)
b. Married/In a Civil Partnership
c. Separated
d. Widowed
e. Co-habitation/Domestic Partnership
f. Prefer not to answer

31. Number of children:

a. 0
b. 1-2
c. 3+

32. Are you the primary caregiver for a child or adult? (more than one answer allowed)

a. Primary carer of a child still necessitating daily support

b. Primary carer of a child 14-18 years old, mostly independent
c. Primary carer or assistant for an older person or people (65 years and over)
d. Primary carer of a child of > 18 years
e. None of the above

33. Who is primarily responsible for childcare outside of school hours

a. Not applicable
b. You
c. Spouse/partner
d. Both you and spouse/partner
e. Family members (grandparents, sister, brother etc.)
f. Paid/non-family
g. Other

34. How do you think/experienced childbearing will/did affect your professional life?

a. Training will take/took longer due to pregnancy/parental leave
b. Had to stop working/training due to pregnancy
c. Will postpone/postponed pregnancy to a later time
d. Will have/had the opportunity to use the time for academic work

35. How would you rate the level of influence of the following items as potential barriers for women in surgery in your country? (Slider bar scale)

e. Bias that women are less interested in surgery
f. Bias that women have less surgical attitude
g. Bias that women are physically less resilient
h. Bias that women are psychologically less suited to be surgeons
i. Discrimination by male colleagues
j. Discrimination by female colleagues
k. Discrimination by patients
l. Unequal treatment from hospital administration with regard to payment/working position
m. Lack of good childcare alternatives
n. Unequal division of household duties
o. Lack of female role models in surgery
p. Other

36. Please rate the following in terms of their potential positive impact to improve women`s career entry in surgery? (Slider bar scale)

a. Option for reduced or flexible working hours
b. Access to onsite childcare support.
c. Access to formal professional development opportunities
~~d.~~ Training and resources to face psychological pressure/burnout
e. Salary equity

f. More opportunities for clinical innovation
g. More role models/females in practice
h. Formal mentoring programs

37. To what extent do you have access to or utilize the following for support in your career? (Slider bar scale)

a) Regular mentor meetings
b) Women surgeon network
c) Social Media
d) Peer to peer mentoring
e) Institutional "assigned" mentor
f) Other: ___________________

38. How often do you feel your gender has influenced your interactions negatively with others in your professional environment?

**Very much**	**Somewhat**	**Not much**	**Not at all**	**Undecided**

39. Please state 3 things that would most improve your work place (with likert scale)

a. Formal Mentorship programme
b. Formal Pastoral/Psychology Resources
c. Parental leaves schemes
d. Protected Academic Time
e. Childcare in-Hospital initiatives
f. Free Text

Thank you for taking the time to do this important survey.
