# Supplementary material for: The impact of gender bias in cardiothoracic surgery in Europe: a European Society of Thoracic Surgeons and European Association for Cardio-Thoracic Surgery survey
Source: Eur J Cardiothorac Surg. 2022 Jan 29;61(6):1390–9. doi: 10.1093/ejcts/ezac034 (PMC9746891; doi:10.1093/ejcts/ezac034)
Supplement: ezac034_supplementary_data [file ezac034_supplementary_data.zip › UNMARKED V10RR ESTS EACTS Survey on Gender Bias_22607.jk.docx]

**Title: The impact of Gender Bias in Cardiothoracic Surgery in Europe: a European Society of Thoracic Surgeons (ESTS) and European Association for Cardio-Thoracic Surgery (EACTS) Survey**

**Authors & Affiliation:**

**Cecilia Pompili^1,2^, Isabelle Opitz^3^, Leah Backhus^4^, Gunda Leschber^5^, Giulia Veronesi^6^, Olivia Lauk^3^, Nuria Novoa^7^, Niccolo’ Daddi^8^, Indu Deglurkar^9^, Julie Cleuziou^10^, Anna Lena Emrich^11^, Francesca D’Auria^12^, Jolanda Kluin^13^**

1.Section of Patient Centred Outcomes Research (PCOR), University of Leeds, UK. 2.Leeds Teaching Hospital NHS Trust, UK 3. University Hospital, Zurich, CH; 4. Stanford University Hospital, Palo Alto, USA; 5. Berlin, DE; 6. Ospedale San Raffaele, Milan, IT 7. University Hospital Salamanca, SP; 8. IRCCS Azienda Ospedaliera Universitaria di Bologna, IT 9. University Hospital of Wales, Cardiff, UK; 10. German Heart Center Munich, DE; 11. Medical University of South Carolina, Charleston, USA and University Medical Center Mainz, DE; 12. San Giovanni di Dio Ruggi d’Aragona University Hospital of Salerno - Schola Medica Salernitana, Salerno, Italy; 13. Academic Medical Center Amsterdam, NE

**Word count: 5277**

**Corresponding Author:**

Cecilia Pompili

Section of Patient Centred Outcomes Research,

University of Leeds

[c.pompili@leeds.ac.uk](mailto:c.pompili@leeds.ac.uk)

St James’ Institute of Oncology, Beckett Street, Leeds LS9 7TF. UK

Telephone: (+44) 0113 20 68939, Fax: (+44) 0113 2067438

**Meeting Presentation**

This work has been presented at the 29^th^ European Society of Thoracic Surgeons (ESTS) Virtual Conference.

**Visual Abstract**

Key question

Are cardiothoracic surgeons in Europe experiencing gender bias during their career?

Key findings

Out of 1118 respondents, 67% of women reported that they experienced being unfairly treated due to gender discrimination.

Take-home message

In-hospital childcare arrangements, formal mentorship and protected academic time were identified as factors which will improve surgical workplace.

**Abstract**

**Objectives**

The European Society of Thoracic Surgeons (ESTS) and the European Association for Cardio-Thoracic Surgery (EACTS) designed a questionnaire to assess the impact of gender bias on a cardiothoracic surgery career.

**Methods**

A 46-item survey investigating gender bias was designed using online survey software from December 2020 to January 2021. All ESTS and EACTS members and non-members included in the mailing lists were invited to complete an electronic survey. Descriptive statistics and comparison between gender groups were performed.

**Results**

Our overall response rate was 11.5% (1118 out of 9764), of which 36.14% were female and 63.69% male. Women were more likely to be younger than men (p<0.0001).66% of women reported having no children compare to only 19% of men (p<0.0001). Only six percent of female versus 22% of males were professor. More women (72%) also reported never having been a formal mentor themselves as compared to men (38%, p<0.0001). 35% of female respondents considered leaving surgery because of episodes of discrimination, compared to 13% of men and 67% of women that they experienced being unfairly treated due to gender discrimination. 31% of male surgeons reported to be very satisfied in their career, compared to only 17% of women (p<0.0001).

**Conclusions**

Women in cardiothoracic surgery report significantly high rates of experiences with bias that may prevent qualified women from advancing in positions of leadership. Efforts to mitigate bias and support the professional development of women are at the centre of the newly formed European Committees.

**Keywords:**

Cardiothoracic surgery; thoracic surgery; gender bias; female leadership; mentorship; professional life

**Introduction**

Despite the increasing proportion of women applying in European Medical schools, there are relatively few women in leadership positions, and several recent publications have highlighted many factors that could contribute to gender inequality in cardiothoracic surgery [1, 2]. However, there is some data to support a change in this landscape. As demonstrated in the US, targeted efforts to support women surgeons have been linked to success evidenced by improved retention and reduced attrition along the training pipeline in cardiothoracic surgery [3]. And most recently, there has been increased interest among national and international societies to address the gender disparity at higher institutional levels [4, 5]. In Europe, this may be a more complicated process, considering difference in cultures, healthcare systems and training programmes but nonetheless is an effort worth doing [6].

The European Society of Thoracic Surgeons (ESTS) and the European Association for Cardio-Thoracic Surgery (EACTS) designed a questionnaire to assess surgeon demographics and the impact of gender bias on a cardiothoracic surgery career.

The findings represent a snapshot of the members of the two Societies. The results of this survey will help in identifying possible initiatives to support next generations in pursuing a cardiothoracic surgery career.

**Material and Methods**

**Ethics Statement**

All ESTS (N:1422) and EACTS (N:8339) members and non-members included in the mailing lists of the Societies that expressed their consent to be contacted, received an email inviting them to complete an electronic survey. The survey was open from the December 4, 2020, to January 17, 2021. Responses were anonymous and collected through a link to a commercially available platform (www.surveymonkey.com). This study was approved by both ESTS and EACTS Councils. Two reminders were sent during this period via email prior to study closure.

**Survey Design**

A 46-item survey investigating gender bias was designed using online survey software and distributed with an introductory letter explaining the purposes of the survey.

There were no exclusion criteria. Social media campaign was also implemented to disseminate the survey (Twitter and LinkedIn) and improve response rate.

All responses were voluntary and anonymous.

The questionnaire was designed by a team of fourteen cardio-thoracic surgeons belonging to both societies (80% females and 20% males) and subsequently submitted for revisions and approvals to the ESTS (11/14 male) and EACTS (12/13 male) Board of Directors. The developer team had experience in questionnaire design methodology and the number and length of the questions was based on agreement.

The questionnaire was designed to elicit objective data regarding the respondents’ demographics, training, and professional information, personal and/or family status (carer’s responsibilities, children), parental leave availability, career choice decisions, perception of the specialty regarding gender bias, access to leadership position and other discriminative factors that may have affect the respondents’ careers.

Participants were asked on a 5-point Likert-like scale to agree or disagree with various statements regarding their role of gender in various well-known difficult scenarios or perceived situations. Statements composed of influence on potential barriers for women in surgical career were also rated on a Likert-like scale.

**Statistical Analysis**

Normal distribution of numeric variables was assessed by the Shapiro–Wilk test. Numeric variables with normal distribution were compared using the unpaired t-test, whereas those without normal distribution were compared using the Mann–Whitney U-test. Categorical variables were compared using the chi squared test or Fisher’s exact test (if the number of observations was <5).

Categorical data were expressed as counts and percentages. Statistical analyses were performed using Stata (Stata Corp, College Station, Texas) with significance at an alpha level of 0.05.

**RESULTS**

***Participant Demographics***

Our overall response rate was 11.5% (1118 out of 9764). Of the 1118 total survey respondents 36.14% were female and 63.69% male (Table 1). Most of the respondents practiced cardiothoracic surgery in an academic hospital/university or Government medical centre setting. Society membership among respondents was 60% declaring a primary EACTS membership compared to 35% declaring primary ESTS membership. Geographic location is reported in Figure 1.

Women in cardiothoracic surgery were more likely to be younger than men(p<0.0001) most of them reporting to be between 30 to 39 years of age. While age distribution among men was spread equally between 30 to 69 years. A similar trend was also observed in terms of number of years post-training, with most female (34%) respondents being in their first 5 years after completion of training compared to 24% of male respondents being 20-29 years out from training (p<0.0001). Ninety-three percent of women reported working full time.

Accordingly, most women were currently in training positions or fellowships compared to men being in their consultant/staff roles. A little bit over eight percent of female as well as male respondents were assistant professors, whereas only six percent of female versus 22% of males were professor. A higher number of male participants (43%) reported to be head of the department compared to women. More male participants had experiences of working outside their countries compared to women (69% vs 53%, p<0.0001).

When asked about personal life, most of the women reported being married or in a civil partnership (47%) while 37% reported being single compared to 84% of men who reported being married. Interestingly 66% of women reported having no children compare to only 19% of men (p<0.0001). This affects the answers of the questions regarding carer’s responsibilities: 37% of male and 25% of female respondents reported to be the primary carer of a child under 18 years of age.

***Mentorship and professional experience***

More female surgeons reported not having had a formal mentor (60%) compared to male respondents (44%, p<0.0001). More women (72%) also reported never having been a formal mentor themselves as compared to men (38%, p<0.0001). There was also a significant difference for the proportion of respondents reporting having participated in formal leadership or mentorship programmes (women: 37% vs men: 56%, p<0.0001). In terms of academic output and grand funding submissions, female respondents reported lower rates compare to the male counterparts.

***Gender bias in professional life***

Thirty-five percent of female respondents indicated that they considered leaving surgery because of episodes of discrimination, compared to only 13% of men (Table 2). Sixty-seven percent of women indicated that they experienced being unfairly treated due to gender discrimination, compared to only 2.5% of male respondents. Few men reported postponing childbearing (14%) compared to nearly half of women respondents despite their younger reported age (44%). Respondents were asked to rate their level of satisfaction in their professional careers. More than one third of male surgeons (31%) reported to be very satisfied, while women reported much less satisfaction (17%, p<0.0001). The question ‘How often do you feel your gender has influenced your interactions negatively with others in your professional environment?’ was answered with ‘very much’ in 24% and with ‘somehow’ by 44% of women whereas the corresponding percentages in answers by male surgeons were 1 and 13 respectively. Fifty-two percent of male answered the aforementioned question with ‘not at all’ compared to only 7% of female surgeons. Interestingly, women declared to feel less valued in their current work environment compared to male (p<0,0001).

***Gender Bias***

Participants to the survey were asked to reflect on the extent of gender bias within our discipline (Tab 3).

The responses from women in cardiothoracic surgery indicated that taking time off for parental leaves for women is still considered an issue in cardiothoracic surgery. Although male and female cardiothoracic surgeons’ responses were more concordant when some of more generic challenges relating to female work-life integration (ie, both agreed with “Female surgeons incur more disadvantages by having a family than male surgeons” and “Some surgeons do not understand the difficulty of female surgeons have balancing work and family/personal life”), they responded markedly differently to most of the other more specific aspects (ie, “a female surgeon can expect resentment if she takes maternity leave” or “Most surgeons in leadership are supportive of female surgeons who want to balance their family and career lives”).

Women more strongly disagree that most of the surgeons would consider a female chairperson a supportive and comfortable figure, compared to the men.

Furthermore, responses from men and women diverged vastly with respect to workplace treatment (eg, “Informal conversations following a meeting often exclude female colleagues,” or “Male and female surgeons have equal income”).

In addition, substantial differences in answering between women and men are seen when asked to identify the potential barriers for women in surgery (Fig 2). More than half of the women indicated that every item except discrimination by female colleagues could be a potential barrier for women in surgery, whereas male rated all items to be less important barriers. Women not only identified discrimination within the surgical field or institutions as potential barriers but also discrimination by patients.

When participants were asked to identify three main factors which will improve their workplace, in-hospital childcare arrangements, formal mentorship and protected academic time were found to play a significant role (Fig 3).

**DISCUSSION**

Women in cardiothoracic surgery report significantly high rates of experiences with bias that may prevent qualified women from advancing in positions of leadership. Poor gender diversity at the leadership level also has negative consequences on the quality of work being done by national and international organizations. Our survey clearly demonstrated that across the two main European cardiothoracic associations, there is still lower percentage of women in leadership or positions of influence. While some of this may be influenced by sampling bias with the majority of female respondents being at a more junior level compared to the male respondents, this is also indicative of the fact that women remain clustered at the junior ranks of most institutions to which they belong. The results confirm the hypothesis that women and men have significant differences with regards to their lived experiences and that some of these differences translate into higher attrition for women. Most of the women surveyed experienced gender discrimination and reported to have thought to leave our speciality due to it.

The Society of Thoracic Surgeons (STS) and Women in Thoracic Surgery (WTS) recently surveyed and obtained responses from 633 members to investigate the extent of gender bias within our discipline[1]. Similarly, to our results, they found that the perception of gender bias varied greatly between male and female respondents. The role of societies like the WTS are groundbreakingly recognized and have already demonstrated their potentials in providing mentorship, training, and networking opportunities for female surgeons. The recently formed ESTS Women in General Thoracic Surgery Committee (WGTS) and EACTS Women in Cardiothoracic Surgery Committee will lead the campaign to inspire more women to fulfil their surgical ambitions.

Furthermore, from US we have also seen promising examples of positive changes. These changes have led to an increased representativity among the Southern Thoracic Surgical Association [7] and also to higher career progression’s success rates of the WTS scholarship awardees[3], confirming the key role of these initiatives to enhance and support careers ‘progression.

The role of mentorship and sponsorship of women, by women, is critically important in achieving maximum career potential, as demonstrated in recent papers in surgical specialities [8]. This has a crucial role in countries where there is a lack of exposure by women from earliest stages of their careers to female role models or mentors [9]. European Societies can play an important role where women who commence a career might subsequently become discouraged and abandon it, unless positively supported. A recent review of mentoring programs and scholarships sponsored by the WTS demonstrated that participation in these structured program was associated with successful pursuit of career milestones at significantly higher rates likely due to fostering of a supportive community for women trainees [3].

The percentage of full professors in surgery who are women is increasing at a rate disproportionately slower than the increases in female medical students and surgery residents [10]. In Europe, this situation may be very heterogeneous, as each country has not only a specific health care system but culturally, the barriers to female progression can be enhanced and strengthened by stereotypes and lack of role models even outside medicine [6, 11-13].

Gender-based disparities in academic surgery have become the focus of recent surveys and articles; however, our overall knowledge of the situation of females in surgery actively involved in the two main European cardiothoracic societies, is still quite limited. Our results showed that the gender disparity is more pronounced in academic medicine and especially when specific output results or grant applications are involved. The early exposure to research in medical school and residency has again a strong potential: a larger US national cohort study showed that the relation between participation in research during residency and future faculty appointment was stronger among women than among men [14]. There is an increasingly defined role of social media to facilitate professional networking and academic dissemination especially in women in thoracic surgery who lack of direct same-sex mentorship exposure [15]. We may not rule out that this has played a role in the favourable improvement in female representation of authorship in our field [16].

Our study findings confirmed how complex is still the position of women in cardiothoracic surgery in Europe, demonstrating the need for education and change. First, most sources of perceived discrimination were gender-specific, which seem to be reflected on the lower level of satisfaction in the professional career for women and a clear tendency of female respondent to consider postponing childbearing to a later time. The changing policies in many countries towards men’s involvement in early child-rearing have received little attention but should be emphasised. New family-friendly policies that encourage men as well as women to take career breaks, or work part-time to share childcare, could have an indirect effect of encouraging their female partners to enter, and remain in, an academic career.

Furthermore, it also clear that male’s consideration of potentials impact of gender bias differs consistently from what women reported. Discrimination from patients is mostly reported by female respondents: as reported across specialities, women physicians are often addressed as Nurse instead of Doctor or are introduced by their first name rather than their title [17]. Societal awareness may play an important role in reducing gender bias inside hospitals. Empowering women from the beginning of their medical school through mentorship programmes or fellowships, may help reducing the misconception reported in our findings that women are less interested, have less surgical attitude or are physically less resilient than men.

Our findings indicate that salary equity for female surgeons, access to onsite childcare support, and more role models’ examples should have a potential positive impact to improve women’s career entry in surgery.

This work will expand the evidence behind the recent implementation of diversity and inclusion initiatives and educational activities in Europe. Research programs and grant institutions equally increased the awareness by forming gender bias committees putting their attention specifically to this topic. Surgical Societies like ESTS and EACTS have formed Women in Thoracic and Cardiothoracic Surgery Committees to ensure and support a climate of respect and inclusion between members.

**Limitations**

This study has some potential limitations which need to be considered interpreting the results.

The survey has involved several countries beyond the European borders, we may not rule out that different socio-economic situations and healthcare system have had an impact on the participants’ responses. Our results may have been affected by the difference of the two groups (male and female) in terms of age: however, this is reflecting the glass ceiling effect that we are still observing in our speciality, confirmed by the difference in women representation in the ESTS trainee members compare to the active ones (44% Vs 19%).

Although our response rate is consistent with other electronic health-care professionals surveys, it is low. Both study length and the fact that it was sent electronically likely contributed to our low response rate. We did not have the data about delivery status of these two major mailing lists or the actual membership status of the recipients, making the estimate of the sample size and response rate limited.

Furthermore, to our knowledge, there are not validated surveys which are investigating specific gender bias in surgery. Recall bias is also a relevant limitation, particularly considering the percentage of senior participants to our survey.

Lastly, we left the definition of ‘formal mentor’, ‘formal leadership and mentorship programme’ and ‘unfairly treated’ to the members themselves.

**Conclusions:**

Our findings depicted a sobering situation in terms of representation of women in cardiothoracic surgery but also when gender bias are considered. Efforts to mitigate bias and support the professional development of women are at the centre of the newly formed European Committees.

**Acknowledgment**

The authors would like to thank all the colleagues who participated to the survey.

**Conflict of Interest Statement**

Conflict of Interest statement: CP reports consultancy for Medela, AZ and BD outside the submitted work. GV reports honoraria from Ab Medica, Intuitive for consultation and proctoring, outside the submitted work. IO reports Roche and AZ: Speakers Bureau, Advisory Board for AZ and MSD and Institutional Grant from Roche and Medtronic outside the submitted work. GL reports AZ: Speakers Bureau outside the submitted work.

**Data Availability Statemen:** All relevant data are within the manuscript and its Supporting Information files.

**Figure Legend**

**Central Image:** Responses by gender to statement about potential experience of biases in the professional career.

**Fig 1:** Geographical location of the respondents

**Fig 2:** Stacked plots of responses by women and men in cardiothoracic surgery to statements about potential barriers to career progression.

**Fig 3:** Stacked plots of responses by both women and men in cardiothoracic surgery to factors which will mostly improve their workplace.

**Tables**

**Table 1: Demographics of respondents**

|  | Total | | Female | | Male | | P value |
| --- | --- | --- | --- | --- | --- | --- | --- |
|  | % | N | % | N | % | N |  |
| **Gender** |  |  |  |  |  |  |  |
| Female | 36.14% | 404 |  |  |  |  |  |
| Male | 63.69% | 712 |  |  |  |  |  |
| Other | 0.18% | 2 |  |  |  |  |  |
| **Professional membership** |  |  |  |  |  |  |  |
| ESTS | 34.79% | 389 |  |  |  |  |  |
| EACTS | 60.38% | 675 |  |  |  |  |  |
| STS | 19.23% | 215 |  |  |  |  |  |
| AATS | 8.59% | 96 |  |  |  |  |  |
| Other (please specify) | 33.90% | 379 |  |  |  |  |  |
| **Current Practice Setting** |  |  |  |  |  |  |  |
| Private – Hospital employed | 14.31% | 160 |  |  |  |  |  |
| Private – Other | 3.22% | 36 |  |  |  |  |  |
| Private – Solo practice | 2.15% | 24 |  |  |  |  |  |
| Academic Clinical (primary) | 42.40% | 474 |  |  |  |  |  |
| Academic Research (primary) | 3.40% | 38 |  |  |  |  |  |
| Government run Hospital | 31.31% | 350 |  |  |  |  |  |
| Other (please specify) | 3.22% | 36 |  |  |  |  |  |
| **Years of Training** |  |  |  |  |  |  |  |
| 1 | 3.76% | 42 |  |  |  |  |  |
| 2 | 7.42% | 83 |  |  |  |  |  |
| 3 | 10.55% | 118 |  |  |  |  |  |
| 4 | 8.32% | 93 |  |  |  |  |  |
| 5 | 23.97% | 268 |  |  |  |  |  |
| >5 | 45.97% | 514 |  |  |  |  |  |
| **Age** |  |  |  |  |  |  | **<0.0001** |
| Under 30 | 6.44% | 72 | 11.63% | 47 | 3.51% | 25 |  |
| 30-39 | 34.26% | 383 | 54.20% | 219 | 23.03% | 164 |  |
| 40-49 | 23.35% | 261 | 20.54% | 83 | 24.85% | 177 |  |
| 50-59 | 21.02% | 235 | 10.64% | 43 | 26.82% | 191 |  |
| 60-69 | 11.36% | 127 | 2.90% | 12 | 16.15% | 115 |  |
| 70+ | 3.58% | 40 | 0.00% | 0 | 5.61% | 40 |  |
| **Number of years post-training** |  |  |  |  |  |  | **<0.0001** |
| Currently in training | 15.38% | 172 | 27.72% | 112 | 8.42% | 60 |  |
| 0-5 years | 22.00% | 246 | 34.15% | 138 | 15.16% | 108 |  |
| 6-9 years | 11.09% | 124 | 14.35% | 58 | 9.12% | 65 |  |
| 10-19 years | 21.20% | 237 | 14.85% | 60 | 24.85% | 177 |  |
| 20-29 years | 17.71% | 198 | 6.43% | 27 | 23.87% | 170 |  |
| ≥30 years | 12.61% | 141 | 2.22% | 9 | 18.53% | 132 |  |
| **Current Position** |  |  |  |  |  |  | **<0.0001** |
| Trainee/Fellow | 23.52% | 263 | 40.59% | 164 | 13.30% | 95 |  |
| Consultant Surgeon | 48.03% | 537 | 36.63% | 148 | 40% | 285 |  |
| Assistant Professor | 8.86% | 99 | 8.41% | 34 | 8.70% | 62 |  |
| Associate Professor | 9.75% | 109 | 6.43% | 26 | 11.37% | 81 |  |
| Professor | 15.56% | 174 | 6.18% | 25 | 21.91% | 156 |  |
| Retired/not currently in practice | 1.16% | 13 | 0.24% | 1 | 1.12% | 8 |  |
| Other (please specify) | 6.08% | 68 | 1.23% | 5 | 0.56% | 4 |  |
| **Primary area of practice (>50%)** |  |  |  |  |  |  | **0.101** |
| Congenital Cardiothoracic Surgery | 8.50% | 95 | 8.90% | 36 | 8.14% | 58 |  |
| Adult Cardiac Surgery | 48.84% | 546 | 45.79% | 185 | 50.70% | 361 |  |
| General Thoracic Surgery | 39.27% | 439 | 40.59% | 164 | 38.48% | 274 |  |
| Other (please specify) | 3.40% | 38 | 4.70% | 19 | 2.66% | 19 |  |
| **Training outside your Country** |  |  |  |  |  |  | **<0.0001** |
| Yes | 63.51% | 710 | 53.40% | 216 | 69.10% | 492 |  |
| No | 36.49% | 408 | 46.53% | 188 | 30.89% | 220 |  |
| **Working pattern** |  |  |  |  |  |  | **0.849** |
| Full time | 94% | 702 | 93% | 270 | 94% | 430 |  |
| Part time | 6% | 47 | 7% | 19 | 6% | 28 |  |
| **Marital status** |  |  |  |  |  |  | **<0.0001** |
| Single (never married or never in a civil partnership) | 16.64% | 176 | 36.87% | 139 | 5.44% | 37 |  |
| Married/In a Civil Partnership | 70.89% | 750 | 47.21% | 178 | 83.90% | 570 |  |
| Separated | 4.16% | 44 | 3.44% | 13 | 4.56% | 31 |  |
| Widowed | 0.95% | 10 | 1.06% | 4 | 0.88% | 6 |  |
| Co-habitation/Domestic Partnership | 6.33% | 67 | 9.54% | 36 | 4.56% | 31 |  |
| Prefer not to answer | 1.04% | 11 | 1.85% | 7 | 0.58% | 4 |  |
| **Number of Children** |  |  |  |  |  |  | **<0.0001** |
| 0 | 35.54% | 376 | 65.78% | 248 | 18.85% | 128 |  |
| 1 | 16.26% | 172 | 12.99% | 49 | 18.11% | 123 |  |
| 2 | 28.07% | 297 | 13.52% | 51 | 36.08% | 245 |  |
| 3 | 13.89% | 147 | 6.36% | 24 | 17.96% | 122 |  |
| more than three | 6.24% | 66 | 1.32% | 5 | 8.93% | 61 |  |
| **Carer responsibility** |  |  | % | N | % | N | **<0.0001** |
| Primary carer of a child still necessitating daily support | 26.18% | 277 | 22% | 82 | 28% | 193 |  |
| Primary carer of a child 14-18 years old, mostly independent | 7.28% | 77 | 3.44% | 13 | 9.42% | 64 |  |
| Primary carer or assistant for an older person or people (65 years and over) | 5.01% | 53 | 7.16% | 27 | 3.82% | 26 |  |
| Primary carer of a child of > 18 years | 7.84% | 83 | 3.71% | 14 | 10% | 69 |  |
| None of the above | 53.69% | 568 | 64% | 241 | 48% | 327 |  |
| **Leadership Position held** |  |  |  |  |  |  |  |
| Lead or Head of Department | 31.38% | 332 | 11% | 40 | 43% | 291 | <0.0001 |
| President of a Cardiothoracic Society | 7.84% | 83 | 3.1% | 12 | 10% | 71 | <0.0001 |
| Chair of an Organization/body | 14.37% | 152 | 7.7% | 29 | 18% | 123 | <0.0001 |
| Executive Committee of a Society/ Association | 17.30% | 183 | 7.9% | 30 | 23% | 153 | <0.0001 |
| Board member in your organization | 23.44% | 248 | 12% | 46 | 30% | 202 | <0.0001 |
| Research Director | 12.10% | 128 | 5% | 19 | 16% | 108 | <0.0001 |
| Trainee Lead | 26.84% | 284 | 19% | 71 | 31% | 213 | <0.0001 |
| None of the above | 38.00% | 402 | 60% | 228 | 26% | 174 | <0.0001 |
| Other leadership roles (please specify): | 7.09% | 75 | 6.6% | 25 | 7.3% | 50 | 0.59 |
| **Have you been a formal mentor?** |  |  |  |  |  |  | **<0.0001** |
| Yes | 49.62% | 525 | 28% | 105 | 62% | 417 |  |
| No | 49.91% | 528 | 72% | 266 | 38% | 259 |  |
| **Have you had a formal mentor?** |  |  |  |  |  |  | **<0.0001** |
| Yes | 50.85% | 538 | 40% | 123 | 56% | 381 |  |
| No | 49.43% | 523 | 60% | 224 | 44% | 296 |  |
| **How many peer-reviewed first or last/senior author publications do you have to your credit?** |  |  |  |  |  |  | **<0.0001** |
| 0-5 | 44.57% | 369 | 62% | 201 | 33% | 168 |  |
| 6-10 | 17.75% | 147 | 19% | 60 | 17% | 86 |  |
| 11-20 | 11.59% | 96 | 9% | 29 | 13% | 67 |  |
| 21-50 | 11.11% | 92 | 4% | 13 | 16% | 78 |  |
| >50 | 14.98% | 124 | 6% | 21 | 21% | 103 |  |
| **Have you applied for external grant funding for research as a Principal Investigator? If so, how many times:** |  |  |  |  |  |  | **<0.0001** |
| 3 or less | 29.71% | 246 | 27.50% | 89 | 31% | 156 |  |
| Over 3 | 16.06% | 133 | 9.50% | 31 | 20% | 102 |  |
| Never | 54.23% | 449 | 63% | 204 | 49% | 244 |  |

**Table 2:** Discrimination’s experiences

|  | Total | | Female | | Male | |  |
| --- | --- | --- | --- | --- | --- | --- | --- |
| **Have you ever considered leaving surgery because of discrimination?** | **%** | N | % | N | % | N | **<0.0001** |
| Yes often | 6.64% | 55 | 13% | 41 | 3% | 14 |  |
| Yes sometimes | 21.62% | 179 | 35% | 114 | 13% | 65 |  |
| No | 51.57% | 427 | 41% | 133 | 58% | 293 |  |
| Not relevant - I have not experienced any discrimination | 18.84% | 156 | 9% | 30 | 25% | 125 |  |
| I don't know | 1.33% | 11 | 2% | 6 | 1% | 5 |  |
| **Did you experience a scenario where you have been unfairly treated due to one of the following** |  |  |  |  |  |  |  |
| Personal Bias | 40.94% | 339 | 47% | 151 | 37% | 188 | <0.0001 |
| Gender discrimination | 27.66% | 229 | 67% | 216 | 2.5% | 13 | <0.0001 |
| Race discrimination | 8.33% | 69 | 9.25% | 30 | 7.76% | 39 | 0.19 |
| No | 38.16% | 316 | 18% | 59 | 45% | 255 | <0.0001 |
| Prefer not to answer | 4.47% | 37 | 3.39% | 11 | 5.17% | 26 |  |
| **How do you think/experienced childbearing will/did affect your professional life?** |  |  |  |  |  |  | **<0.0001** |
| Training will take/took longer due to pregnancy/parental leave | 42.15% | 349 | 30% | 97 | 50% | 251 |  |
| Had to stop working/training due to pregnancy | 15.58% | 129 | 16% | 53 | 15% | 76 |  |
| Will postpone/postponed pregnancy to a later time | 25.97% | 215 | 44% | 143 | 14% | 71 |  |
| Will have/had the opportunity to use the time for academic work | 16.30% | 135 | 10% | 31 | 21% | 104 |  |
| **Please indicate level of satisfaction in your professional career** |  |  |  |  |  |  | **<0.0001** |
| Very satisfied | 25.97% | 215 | 17% | 56 | 31% | 158 |  |
| Satisfied | 47.95% | 397 | 47% | 153 | 48% | 243 |  |
| Neither satisfied nor dissatisfied | 15.94% | 132 | 19% | 60 | 14% | 72 |  |
| Dissatisfied | 8.45% | 70 | 14% | 45 | 5% | 25 |  |
| Very dissatisfied | 1.69% | 14 | 3% | 10 | 0.80% | 4 |  |
| **How often do you feel your gender has influenced your interactions negatively with others in your professional environment?** |  |  |  |  |  |  | **<0.0001** |
| Very Much | 10.39% | 86 | 24% | 79 | 1.39% | 7 |  |
| Somehow | 25.12% | 208 | 44% | 142 | 13% | 66 |  |
| Not Much | 26.09% | 216 | 23% | 74 | 28% | 142 |  |
| Not at All | 34.78% | 288 | 7.09% | 23 | 52% | 263 |  |
| Undecided | 3.62% | 30 | 1.80% | 6 | 4.78% | 24 |  |
| **How valued do you feel in your current work environment?** |  |  |  |  |  |  | **<0.0001** |
| Extremely valuable | 16.06% | 133 | 10% | 33 | 20% | 100 |  |
| Very valuable | 39.13% | 324 | 31% | 99 | 45% | 224 |  |
| Somewhat valuable | 33.45% | 277 | 41% | 133 | 28% | 143 |  |
| Not so valuable | 8.09% | 67 | 13% | 43 | 4.78% | 24 |  |
| Not at all valuable | 3.26% | 27 | 4.93% | 16 | 2.19% | 11 |  |

**Table 3:** Table of responses by women and men in cardiothoracic surgery to gender bias statements.

|  | Male % | Female% |
| --- | --- | --- |
| A female surgeon can expect resentment if she takes parental leave | | |
| Agree/strongly agree | 28 | 63 |
| Disagree/strongly Disagree | 41 | 16 |
| Neutral | 31 | 21 |
| Most surgeons in leadership are supportive of female surgeons who want to balance their family and career lives | | |
| Agree/strongly agree | 47 | 14 |
| Disagree/strongly Disagree | 26 | 68 |
| Neutral | 28 | 18 |
| Surgeons who bring up issues about balancing family and career usually would be supported | | |
| Agree/strongly agree | 48 | 55 |
| Disagree/strongly Disagree | 25 | 22 |
| Neutral | 27 | 23 |
| Most surgeons would feel comfortable and supportive of a female chairperson | | |
| Agree/strongly agree | 51 | 22 |
| Disagree/strongly Disagree | 17 | 56 |
| Neutral | 32 | 22 |
| Informal conversations following a meeting often exclude female colleagues | | |
| Agree/strongly agree | 8 | 37 |
| Disagree/strongly Disagree | 77 | 40 |
| Neutral | 16 | 23 |
| Male and female surgeons have equal income | | |
| Agree/strongly agree | 64 | 30 |
| Disagree/strongly Disagree | 18 | 55 |
| Neutral | 18 | 15 |
| Male surgeons are as likely to discuss academic issues with a female colleague. | | |
| Agree/strongly agree | 70 | 36 |
| Disagree/strongly Disagree | 10 | 30 |
| Neutral | 20 | 34 |
| Some surgeons do not understand the difficulty female surgeons have balancing work and family/personal life. | | |
| Agree/strongly agree | 70 | 84 |
| Disagree/strongly Disagree | 14 | 6 |
| Neutral | 16 | 10 |
| A male surgeon can expect resentment if he takes parental leave. | | |
| Agree/strongly agree | 43 | 36 |
| Disagree/strongly Disagree | 30 | 39 |
| Neutral | 27 | 25 |
| Female surgeons incur more disadvantages by having a family than male surgeons. | | |
| Agree/strongly agree | 67 | 88 |
| Disagree/strongly Disagree | 17 | 6 |
| Neutral | 16 | 5 |
| Female surgeons who have taken time off to have children are considered just as committed as those who have not taken time off. | | |
| Agree/strongly agree | 44 | 21 |
| Disagree/strongly Disagree | 29 | 59 |
| Neutral | 27 | 20 |

**Supplementary Data**

Appendix 1: Questionnaire

**Bibliography**

[1] Ceppa DP, Dolejs SC, Boden N, Phelan S, Yost KJ, Edwards M *et al.* *Gender Bias and Its Negative Impact on Cardiothoracic Surgery*. The Annals of thoracic surgery 2020;**109**:14-17.

[2] Ceppa DP, Antonoff MB, Tong BC, Timsina L, Ikonomidis JS, Worrell SG *et al.* *2020 Women in Thoracic Surgery update on the status of women in cardiothoracic surgery*. The Annals of thoracic surgery 2021.

[3] Williams KM, Hironaka CE, Wang H, Bajaj SS, O'Donnell CT, Sanchez M *et al.* *Women in Thoracic Surgery Scholarship: Impact on Career Path and Interest in Cardiothoracic Surgery*. The Annals of thoracic surgery 2020.

[4] Ceppa DP, Ikonomidis JS, Timsina LR, Boden N, Kane LC, Donington JS. *STS 2019 Workforce Report: Ad Hoc Analysis of Women in Cardiothoracic Surgery*. The Annals of thoracic surgery 2021;**111**:383-85.

[5] Stephens EH, Robich MP, Walters DM, DeNino WF, Aftab M, Tchantchaleishvili V *et al.* *Gender and Cardiothoracic Surgery Training: Specialty Interests, Satisfaction, and Career Pathways*. The Annals of thoracic surgery 2016;**102**:200-6.

[6] Pompili C, Veronesi G, Novoa NM, Gomez-Hernandez MT, Sharkey AJ, Bhatti F *et al.* *Women in thoracic surgery: European perspectives*. Journal of thoracic disease 2021;**13**:439-47.

[7] Olive JK, Preventza OA, Blackmon SH, Antonoff MB. *Representation of Women in The Society of Thoracic Surgeons Authorship and Leadership Positions*. The Annals of thoracic surgery 2019.

[8] Bruce AN, Battista A, Plankey MW, Johnson LB, Marshall MB. *Perceptions of gender-based discrimination during surgical training and practice*. Med Educ Online 2015;**20**:25923.

[9] Wang H, Bajaj SS, Williams KM, Heiler JC, Pickering JM, Manjunatha K *et al.* *Early Engagement in Cardiothoracic Surgery Research Enhances Future Academic Productivity*. The Annals of thoracic surgery 2020.

[10] Weiss A, Lee KC, Tapia V, Chang D, Freischlag J, Blair SL *et al.* *Equity in surgical leadership for women: more work to do*. American journal of surgery 2014;**208**:494-8.

[11] Preece R, Ben-David E, Rasul S, Yatham S. *Are we losing future talent? A national survey of UK medical student interest and perceptions of cardiothoracic surgery†*. Interactive cardiovascular and thoracic surgery 2018;**27**:525-29.

[12] Laskar M, Spinosi AM, Bendjebla Y, Moreau J, Dahan M. *Are we running out of thoracic or cardiac surgeons? Demography of thoracic and cardiac surgeons in France in 2012*. Interactive cardiovascular and thoracic surgery 2013;**16**:470-4.

[13] Bellini MI, Graham Y, Hayes C, Zakeri R, Parks R, Papalois V. *A woman’s place is in theatre: women’s perceptions and experiences of working in surgery from the Association of Surgeons of Great Britain and Ireland women in surgery working group*. BMJ open 2019;**9**.

[14] Andriole DA, Jeffe DB. *The road to an academic medicine career: a national cohort study of male and female U.S. medical graduates*. Acad Med 2012;**87**:1722-33.

[15] Luc JGY, Archer MA, Arora RC, Bender EM, Blitz A, Cooke DT *et al.* *Social Media Improves Cardiothoracic Surgery Literature Dissemination: Results of a Randomized Trial*. The Annals of thoracic surgery 2020;**109**:589-95.

[16] Luc JGY, Vervoort D, Percy E, Hirji S, Mann GK, Phan K *et al.* *Trends in Female Authorship: A Bibliometric Analysis of The Annals of Thoracic Surgery*. The Annals of thoracic surgery 2021;**111**:1387-93.

[17] Files JA, Mayer AP, Ko MG, Friedrich P, Jenkins M, Bryan MJ *et al.* *Speaker Introductions at Internal Medicine Grand Rounds: Forms of Address Reveal Gender Bias*. Journal of Women's Health 2017;**26**:413-19.
